# Characterization of the Plastid Genomes of Four *Caroxylon* Thunb. Species from Kazakhstan

**DOI:** 10.3390/plants13101332

**Published:** 2024-05-12

**Authors:** Shyryn Almerekova, Moldir Yermagambetova, Bektemir Osmonali, Polina Vesselova, Saule Abugalieva, Yerlan Turuspekov

**Affiliations:** 1 Institute of Plant Biology and Biotechnology, Almaty 050040, Kazakhstan; almerekovakz@gmail.com (S.A.); ermaganbetova.moldir@bk.ru (M.Y.); absaule@yahoo.com (S.A.); 2 Faculty of Biology and Biotechnology, Al-Farabi Kazakh National University, Almaty 050040, Kazakhstan; 3 Institute of Botany and Phytointroduction, Almaty 050040, Kazakhstan; be96ka_kz@mail.ru (B.O.); pol_ves@mail.ru (P.V.)

**Keywords:** *Caroxylon*, plastid genome, genome comparison, variable regions, phylogenetic relationships, next-generation sequencing

## Abstract

The family Chenopodiaceae Vent. (Amaranthaceae *s.l*.) is known for its taxonomic complexity, comprising species of significant economic and ecological importance. Despite its significance, the availability of plastid genome data for this family remains limited. This study involved assembling and characterizing the complete plastid genomes of four *Caroxylon* Thunb. species within the tribe Salsoleae *s.l*., utilizing next-generation sequencing technology. We compared genome features, nucleotide diversity, and repeat sequences and conducted a phylogenetic analysis of ten Salsoleae *s.l*. species. The size of the plastid genome varied among four *Caroxylon* species, ranging from 150,777 bp (*C. nitrarium*) to 151,307 bp (*C. orientale*). Each studied plastid genome encoded 133 genes, including 114 unique genes. This set of genes includes 80 protein-coding genes, 30 tRNA genes, and 4 rRNA genes. Eight divergent regions (*accD*, *atpF*, *matK*, *ndhF-ndhG*, *petB*, *rpl20-rpl22*, *rpoC2*, and *ycf3*) were identified in ten Salsoleae *s.l*. plastid genomes, which could be potential DNA-barcoding markers. Additionally, 1106 repeat elements were detected, consisting of 814 simple sequence repeats, 92 tandem repeats, 88 forward repeats, 111 palindromic repeats, and one reverse repeat. The phylogenetic analysis provided robust support for the relationships within *Caroxylon* species. These data represent a valuable resource for future phylogenetic studies within the genus.

## 1. Introduction

Chenopodiaceae Vent. (Amaranthaceae *s.l*.) is one of the largest and most ancient plant families inhabiting desert and semi-desert regions worldwide [1,2]. The Chenopodiaceae family encompasses approximately 1700 species distributed among around 110 genera [3]. Ecologically, representatives of the Chenopodiaceae family play crucial roles in desert ecosystems, serving as vital food sources for herbivores and contributing significantly to soil stabilization [4]. One of the family’s largest and most important tribes is Salsoleae *s.l*. [5,6]. The precise number of genera in Salsoleae *s.l*. worldwide remains uncertain, with estimates ranging from 32 to 98 [5]. The species of Salsoleae *s.l*. are widely distributed across desert and semi-desert regions spanning Central Asia, the Middle East, Africa, and Europe [7,8]. Among these members is the genus *Caroxylon* Thunb., formerly classified as a section within the *Salsola* L. before being reinstated as a distinct genus [9]. According to POWO (Plants of the World Online) [10], the genus comprises 128 species globally, with nine of them found in Kazakhstan. These species are economically significant as forage plants and sources of medicinal compounds [4,7].

Numerous studies have been conducted to explore the taxonomy of the tribe, including *Caroxylon* species, employing both morphological characteristics and molecular genetics methodologies [3,6,9,11,12,13,14,15,16,17,18]. However, despite these efforts, a precise taxonomy of the tribe has yet to be established.

Akhani et al. [9] performed an extensive phylogenetic study on the Salsoleae *s.l*. utilizing sequences from the nuclear ribosomal internal transcribed spacer and the plastid *psbB-psbH* region. Certain representatives previously classified under **Salsola* s.l*. have been redistributed among reinstated or recently established genera [9]. For instance, **Salsola* canescens* (Moq.) Boiss., previously included in the *Salsola* sect. *Belanthera* Iljin under the name *S. boissieri* Botsch. [19] was transferred to the genus *Caroxylon* as *Caroxylon canescens* (Moq.) Akhani [9]. However, Sukhorukov et al. [20] indicated uncertainty regarding the taxonomic transfer of *S. canescens* to the genus *Caroxylon*, and they established a new genus, *Akhania*, for this species based on molecular phylogeny using *ITS* and *psbB-psbH* nucleotide sequences. Furthermore, using three DNA barcodes, Wen and co-authors [6] suggested that all species of tribe Salsoleae *s.l*. were composed of three monophyletic subunits: Salsolea *s.str*., the *Kali* clade, and Caroxylonea. Despite the comprehensive phylogenetic study conducted by Akhani et al. [9], not all species from Central Asia, including Kazakhstan, were included in the analysis.

In addition to the numerous phylogenetic analyses conducted on Salsoleae *s.l*. species utilizing nuclear and plastid genome markers, population genetics analysis was also performed. The Amplified Length Polymorphism (AFLP) technique was employed to identify and use AFLP markers to study genetic relationships in four *Salsola* species [21]. Inter simple sequence repeat (ISSR) and start codon targeted (SCoT) molecular markers were used to analyze the genetic relationships between the different species of *Salsola* [22]. Seventeen simple sequence repeat (SSR) markers of *Beta* were employed in the cross-genera amplification of five morphologically distinct invasive *Salsola* taxa [23]. However, only six of them were successfully amplified within the studied *Salsola* taxa [23]. Additionally, several studies were conducted to assess the cross-genera transferability of these SSR markers to *Salsola* species [24,25]. However, there is a notable absence of population structure analyses in representatives of *Caroxylon*.

The SSR markers are valuable genetic markers extensively employed in population studies [26,27,28,29]. These markers, composed of tandemly repeated motifs, offer unique advantages in elucidating genetic variation and evolutionary relationships within and among species [30,31]. While SSRs are widespread across the genomes of diverse organisms [32], cpSSRs specifically reside within the chloroplast genome, primarily found in plant cells [26]. Despite the rising popularity of SSR markers in plant population studies, there is a noticeable absence of research on economically significant *Caroxylon* species.

Plastid genome characterization studies of representatives from the tribe Salsoleae *s.l*. have been limited, with only a few published investigations. Specifically, Li et al. [33] examined **Salsola* abrotanoides*, while Xie et al. [34] investigated *Caroxylon passerinum*. Comparative analysis of plastid genome data helps reconstruct phylogenetic trees and provides valuable information for understanding the evolutionary relationships among plant species [35,36]. The rapid advancement of next-generation sequencing technology has greatly improved the efficiency and accessibility of obtaining complete plastid genome nucleotide sequences [37]. Sequencing plastid genomes in plants is crucial for advancing various fields of research, including evolutionary biology [38], taxonomy [39], biogeography [40], breeding [41], and conservation [42]. However, despite the widespread use of plastid genome data for comparative analysis, no comparative studies have been conducted on *Caroxylon* plastid genomes.

Using the nomenclature based on molecular evidence [6,9], we studied the sequencing, assembly, and annotation of plastid genomes of four *Caroxylon* species collected in Kazakhstan: *C. orientale*, *C. nitrarium*, *C. dzhungaricum*, and *C. laricinum*. These species thrive in rocky and clay soils, serving as essential forage for herbivores during autumn and winter. *C. orientale* and *C. nitrarium* are widely distributed throughout Kazakhstan’s territory. Furthermore, we conducted a comparative analysis to characterize these plastomes, comparing them with related taxa plastomes available in GenBank. The objectives of this study encompassed providing plastid genome data for four newly sequenced *Caroxylon* species; comparing the structure of their plastid genomes and identifying variable regions suitable as potential DNA barcoding markers for species identification and phylogenetic analysis; exploring repeat elements, including simple sequence repeats, tandem repeats, forward repeats, palindromic repeats, and reverse repeats within the analyzed plastomes; and utilizing common protein-coding gene sequences for constructing a phylogenetic tree, aiming to clarify the phylogenetic relationships among the studied species.

## 2. Results

### 2.1. Features of the Plastid Genome

**Figure 1:**
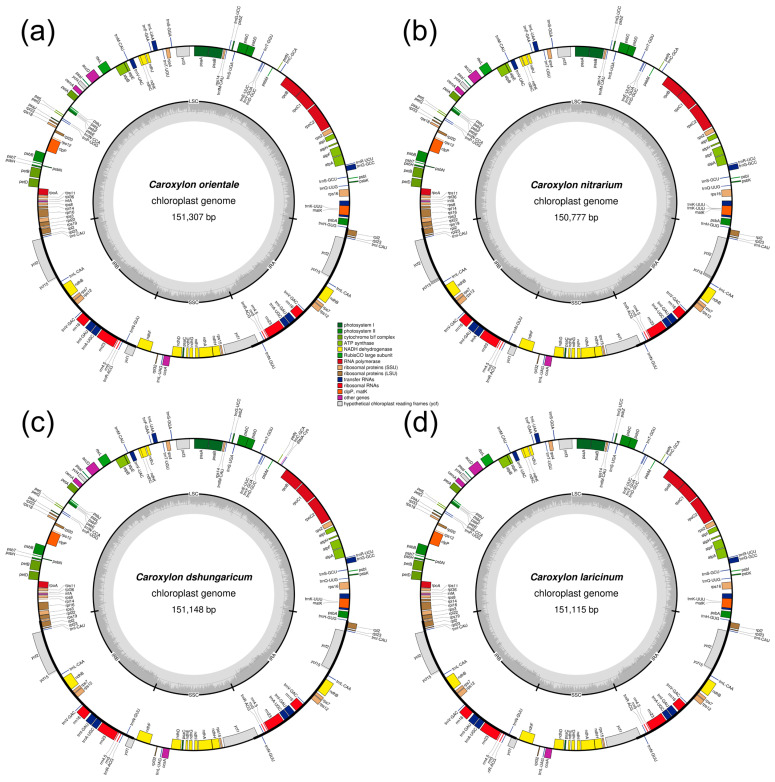
Plastid genome maps of the four *Caroxylon* species. (**a**) *C. orientale*, (**b**) *C. nitrarium*, (**c**) *C. dzhungaricum*, and (**d**) *C. laricinum*. Genes from various functional categories are colorized accordingly. The darker gray on the inner circle represents GC content, while the lighter gray denotes AT content.

In this study, we sequenced plastid genomes of four *Caroxylon* species (*C. orientale*, *C. nitrarium*, *C. dzhungaricum*, and *C. laricinum*) that were collected in Kazakhstan. A total of 27,353,686, 24,456,970, 21,851,266, and 24,687,480 paired-end reads were obtained, each having a sequence length of 151 bp. Subsequently, 25,260,324, 21,693,212, 19,639,670, and 22,024,448 high-quality reads were used for mapping the plastid genome of *C. orientale*, *C. nitrarium*, *C. dzhungaricum*, and *C. laricinum*, respectively. High-quality data with clean reads totaling over 3.8, 3.5, 3.1, and 3.6 Gb were generated for *C. orientale*, *C. nitrarium*, *C. dzhungaricum*, and *C. laricinum*, respectively. The sequencing quality values Q20 were determined to be 97.58%, 96.62%, 96.94%, and 96.8% for *C. orientale*, *C. nitrarium*, *C. dzhungaricum*, and *C. laricinum*, respectively. The Q30 values were 92.11%, 90.07%, 90.76%, and 90.47% for the same species. The newly sequenced plastomes have been submitted to GenBank with the following accession numbers: OR551471 (*C. orientale*), OR552116 (*C. nitrarium*), PP503423 (*C. dzhungaricum*), and PP503424 (*C. laricinum*). The complete plastid genome in four *Caroxylon* species ranged in size from 150,777 bp in *C. nitrarium* to 151,307 bp in *C. orientale* (Table 1). Each of the newly sequenced plastomes exhibited a typical quadripartite structure consisting of four regions: a pair of inverted repeats (IRa and IRb), the large single-copy region (LSC), and the small single-copy region (SSC) (Figure 1). The length of the LSC region varied from 83,329 bp in *C. nitrarium* to 83,706 bp in *C. dzhungaricum*. The SSC region ranged from 18,266 bp in *C. orientale* to 18,999 bp in *C. laricinum*. Additionally, the IR region varied in size from 48,438 bp (*C. laricinum*) to 51,348 bp (*C. orientale*). In terms of GC content, the IR regions displayed the highest GC content, ranging from 42.57% (*C. orientale*) to 42.72% (*C. laricinum*). Following this, the LSC region showed GC content ranging from 34.67% (*C. dzhungaricum*) to 34.69% (*C. laricinum*). Conversely, the SSC region exhibited the lowest GC content, varying from 29.68% (*C. orientale*) to 30.33% (*C. nitrarium*). The overall GC content of the plastome sequences for *C. orientale*, *C. nitrarium*, *C. dzhungaricum*, and *C. laricinum* was 36.69%, 36.84%, 36.68%, and 36.71%, respectively (Table 1).

Each of the *C. orientale*, *C. nitrarium*, *C. dzhungaricum*, and *C. laricinum* plastid genomes encoded 133 genes; 114 of these genes were unique, including 80 protein-coding genes, 30 tRNA genes, and 4 rRNA genes (Table 1). Seven tRNA genes (*trnA-UGC*, *trnI-CAU*, *trnI-GAU*, *trnL-CAA*, *trnN-GUU*, *trnR-ACG*, and *trnV-GAC*), eight protein-coding genes (*rps7*, *rps12*, *rpl2*, *rpl23*, *ndhB*, *ycf1*, *ycf2*, and *ycf15*), and four rRNA genes (*rrn4.5*, *rrn5*, *rrn16*, and *rrn23*) were identified as duplicated within IR regions. Among the 114 unique genes, 17 contain introns: 6 tRNA genes (*trnA-UGC*, *trnG-GCC*, *trnI-GAU*, *trnK-UUU*, *trnL-UAA*, and *trnV-UAC*) and 11 protein-coding genes (*rps12*, *rps16*, *rpl16*, *rpoC1*, *atpF*, *ndhA*, *ndhB*, *petB*, *petD*, *clpP*, and *ycf3*). Notably, *clpP* and *ycf3* stand out as the only genes in this context with two introns each, while the other 15 genes are characterized by having a single intron each (Table 2).

### 2.2. Plastome Analysis by Sliding Window

**Figure 2:**
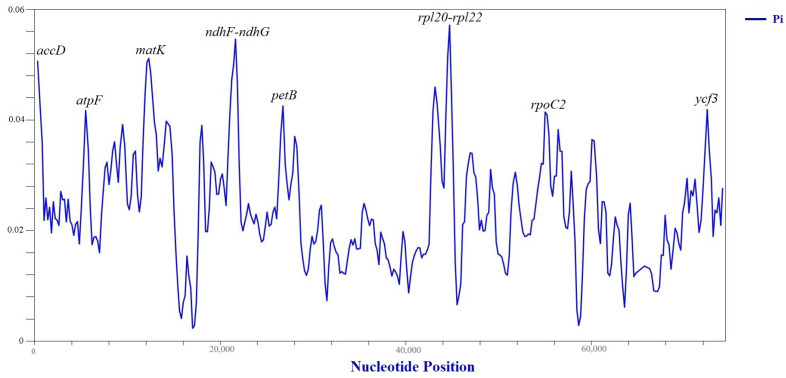
Sliding window analysis of the common protein-coding genes (window length: 600 bp; step size: 200 bp) in plastid genomes of the *Caroxylon* species. The vertical axis indicates the nucleotide diversity for each window, while the horizontal axis denotes the midpoint position.

We utilized DnaSP 6 software to perform a sliding window analysis aimed at determining the nucleotide diversity (Pi) value within the 80 protein-coding genes in plastid genomes of the *Caroxylon* species. Based on the sequence alignment of common protein-coding genes, we identified eight hypervariable regions: *accD*, *atpF*, *matK*, *ndhF-ndhG*, *petB*, *rpl20-rpl22*, *rpoC2*, and *ycf3*. Seven of these regions (*accD*, *atpF*, *matK*, *petB*, *rpl20-rpl22*, *rpoC2*, and *ycf3*) are situated within the LSC region, while only one region (*ndhF-ndhG*) is found in the SSC region (Figure 2). Among the variable regions identified, *rpl20-rpl22* exhibited the highest Pi value at 0.05711 (Table 3).

The nonsynonymous (Ka) and synonymous (Ks) substitutions were calculated using DNASP 6 between studied plastid genomes (Table 3). The results suggested that the ratios of Ka/Ks in six out of ten genes listed in Table 3 were >1, indicating that they were under positive selection.

### 2.3. IR and SC Regions Boundary Analysis

**Figure 3:**
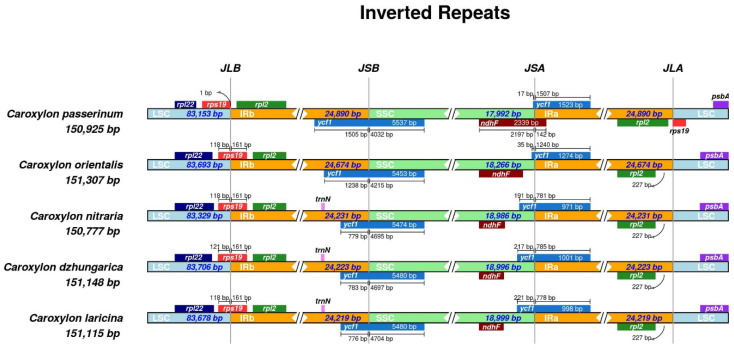
Comparisons of the borders of LSC, IR, and SSC regions among the five plastid genomes JLB (junctions between LSC and IRb regions), JSB (junctions between IRb–SSC regions), JSA (junctions between SSC–IRa regions), and JLA (junctions between IRa–LSC regions).

Our analysis investigated the boundaries of the IR-SSC and IR-LSC regions in the plastomes of four *Caroxylon* species, comparing them with the reference sample, *C. passerinum*. We observed that the gene *rps19* flanked the junction between the LSC and IRb regions in the four *Caroxylon* species, whereas in the reference sample *C. passerinum*, the *rps19* gene, including a pseudogene, was located within the LSC region. Conversely, the *ndhF* gene in *C. passerinum* was positioned to flank the junction between the SSC and IRa regions, while in the four *Caroxylon* samples, this gene was situated within the SSC region. The *ycf1* gene exhibited a consistent pattern across all five samples, spanning the boundaries of the SSC and IRb regions. Additionally, a duplicated copy of the *ycf1* gene was consistently observed at the junction between the SSC and IRa regions in each sample (Figure 3). 

### 2.4. Repeat Sequence Analysis

We identified 814 simple sequence repeats (SSRs) across the four *Caroxylon* plastomes using the MISA tool. SSRs vary among the four *Caroxylon* plastomes, ranging from 198 in *C. nitrarium* to 208 in *C. dzhungaricum*. The analysis revealed that mononucleotide repeats were the most abundant motifs, representing 72.97% of the total SSRs. Dinucleotide repeats followed, accounting for 19.04%, while tetranucleotide repeats constituted 4.18%. Hexanucleotide repeats, representing 0.25% of the total SSRs, were uniquely identified within the *C. laricinum* plastome. The vast majority of mononucleotide repeats consisted of A/T sequences (71.38%), with a minor portion (2.19%) comprising C/G sequences. Regarding dinucleotide repeats, AT/AT repeats were predominant, representing 54.84%, while AC/GT repeats constituted only 6.45%, and AG/CT repeats made up 38.71% (Table 4). Most of the identified SSRs were located in the non-coding and LSC regions of *Caroxylon* plastomes (Supplementary File S1).

**Figure 4:**
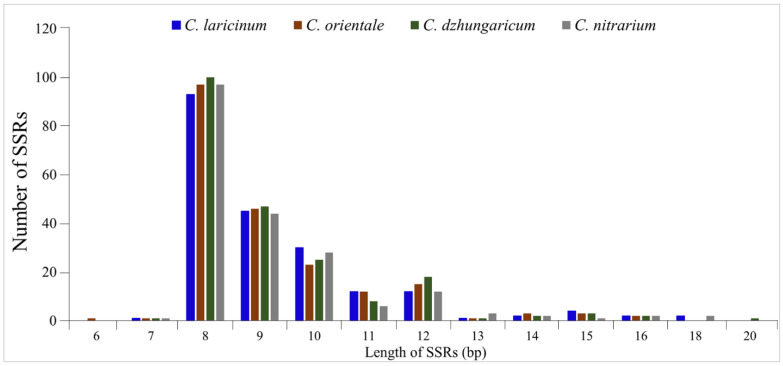
The length of the identified simple sequence repeats in plastomes of *C. laricinum*, *C. orientale*, *C. dzhungaricum*, and *C. nitrarium*.

The lengths of the identified simple sequence repeats varied from 6 bp in *C. laricinum* to 20 bp in *C. dzhungaricum* plastid genomes. The majority of SSRs were eight base pairs in length, with 93, 97, 100, and 97 repeats identified in the plastid genomes of *C. laricinum*, *C. orientale*, *C. dzhungaricum*, and *C. nitrarium*, respectively. The categorization of repeats with different lengths is presented in Figure 4.

**Figure 5:**
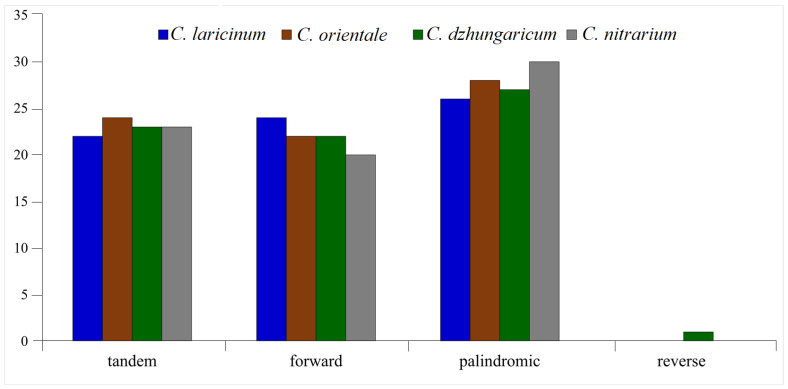
Number of tandem, forward, palindromic, and reverse repeats in the plastomes of the four *Caroxylon* species.

Furthermore, our analysis identified tandem, forward, palindromic, and reverse repeat types in the plastomes of the four *Caroxylon* species. A total of 292 repeats were detected, comprising 92 tandem repeats, 88 forward repeats, 111 palindromic repeats, and just one reverse repeat. The reverse repeat was found solely within the plastome of *C. dzhungaricum* (Figure 5).

### 2.5. Phylogenetic Analysis

**Figure 6:**
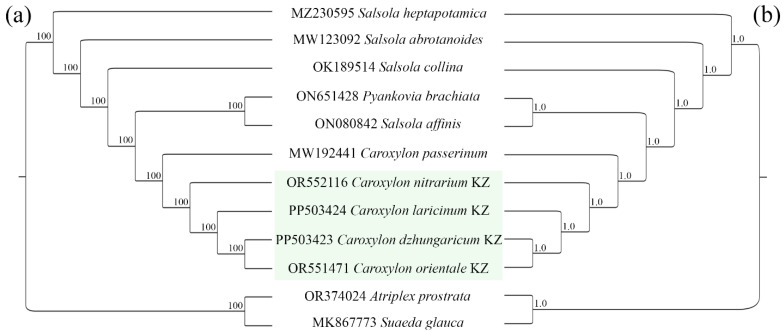
Phylogenetic tree based on nucleotide sequences from protein-coding genes using the Maximum Likelihood (**a**) and Bayesian Inference (**b**) methods. ML bootstrap value and BI posterior probability are given at each node. The species from Kazakhstan were highlighted with the letters “KZ”.

To elucidate the phylogenetic relationships among the ten species from the Salsoleae tribe, we reconstructed the phylogenetic tree using the Maximum Likelihood (ML) and Bayesian Inference (BI) methods. We utilized nucleotide sequences of 80 protein-coding genes, commonly found in 12 chloroplast genomes, including two outgroup species. Two datasets, (1) nucleotide sequences derived from protein-coding genes and (2) nucleotide sequences encompassing the entire plastid genome, were employed to construct the phylogenetic trees. The phylogenetic analyses based on the ML and BI methods grouped all ten samples into a single clade with strong bootstrap support. The species examined in this study (*C. orientale*, *C. nitrarium*, *C. dzhungaricum*, and *C. laricinum*) formed a distinct subclade alongside species obtained from GenBank (*C. passerinum*), thus constituting a subgroup within the *Caroxylon* clade (Figure 6).

The phylogenetic analyses performed on the entire plastid genome sequences revealed results consistent with those obtained from the dataset of protein-coding gene sequences. The ML and BI trees generated from the complete plastid genome sequences are included in Supplementary File S2.

## 3. Discussion

Data from plastid genomes offer valuable insights for taxonomic studies aimed at assessing evolutionary relationships and conducting comparative analyses across various taxonomic levels [43,44,45,46]. In this investigation, we obtained and analyzed four *Caroxylon* plastid genomes using Illumina sequencing technology. The comparative analysis revealed consistent genome structure and gene count across these examined genomes. The study of plastid genomes of *C. orientale*, *C. nitrarium*, *C. dzhungaricum*, and *C. laricinum* revealed a consistent presence of 133 genes in each species, with 114 genes being unique, including 80 protein-coding genes, 30 tRNA genes, and 4 rRNA genes (Table 1).

Compared with the plastome annotation of *C. passerinum* [34], variations were observed in the number of protein-coding and tRNA genes. Specifically, the total number of protein-coding genes in the *C. passerinum* plastome was reported as 89, including duplicated genes. However, in the four newly sequenced plastomes, this count was 88, as the pseudogene *rps19* was not annotated due to its short length. Conversely, while the *trnG-GCC* and *trnK-UUU* genes were not annotated in the *C. passerinum* plastome [34], these genes were identified in the four *Caroxylon* species analyzed in this study, resulting in a total of 37 tRNA genes, including duplicated genes.

Our sliding window analysis investigation further identified eight relatively variable regions, encompassing *accD*, *atpF*, *matK*, *ndhF-ndhG*, *petB*, *rpl20-rpl22*, *rpoC2*, and *ycf3* (Figure 2), where six out of ten genes in those regions were under positive selection (Table 3). Noteworthy among these findings is the recognition of the *matK* gene as a core plant barcode by the CBOL Plant Working Group [47]. Moreover, our examination of nucleotide diversity demonstrated similar variability in other species. For instance, Liu et al. [48] and Almerekova et al. [49] identified variability in the *accD* region among *Quercus* and *Juniperus* species, respectively. Rodda and Niissalo [50] observed variability in the *accD* and *ndhF* regions within *Hoya* species plastomes. Additionally, variability in the *rpl20* region among *Aerides* species’ plastid genome was noted by Chen et al. [51], while Ding et al. [52] reported variability in the *rpl22* region among the *Clethra* plastid genome. The identified relatively variable regions have the potential to serve as molecular markers for phylogenetic studies of *Caroxylon* species.

The boundaries between IR and SC regions are conserved in studied *Caroxylon* species (Figure 3). The IRb/LSC boundary was consistently found within the *rps19* gene among the four newly sequenced species analyzed in this study. Similarly, the IRb/SSC boundary was located within the *ycf1* gene, while the IRa/SSC boundary was identified within the duplicated copy of the *ycf1* gene. These results are in accordance with previous studies conducted on different species [53,54]. Notably, no major changes in the position of IR regions were observed in the studied plastid genomes, suggesting the absence of substantial structural rearrangements [55,56]. Among more closely related species, any observed shifts in IR boundaries tended to be relatively minor [57].

SSRs are widespread throughout plastid genomes across different species and are extensively employed in plant population studies [58,59,60]. In this study, the identified SSRs varied in number, with counts ranging from 198 in *C. nitrarium* to 208 in *C. dzhungaricum* plastomes, resulting in a total of 814 SSRs (Table 4). Similar to the previous studies [43,61], mononucleotide repeats were the most prevalent motifs, comprising 594 of the total identified SSRs. Polyadenine (poly-A) or polythymine (poly-T) repeats were the most abundant in the plastid genome of four *Caroxylon* species collected in Kazakhstan, a common phenomenon observed in the plastid genomes of higher plants [62,63,64]. According to the previous findings [65,66], most of the identified SSRs were located in the non-coding and LSC regions of the four examined plastomes. The SSRs revealed in this analysis offer valuable resources for investigating the population genetics of *Caroxylon* species, thereby filling the gaps in population studies within this genus.

The plastid genome significantly conserves structure and gene composition [57], making it a valuable resource for analyzing phylogenetic relationships across diverse taxonomic levels [67]. Before this study, phylogenetic relationships within the tribe Salsoleae had been assessed using only a limited number of genes, and the precise taxonomy of the tribe remains unresolved. In this analysis, nucleotide sequences from common protein-coding genes of ten representatives of the Salsoleae tribe were employed, comprising four newly sequenced *Caroxylon* species. The phylogenetic tree was reconstructed using the Maximum Likelihood (ML) method. The resulting phylogenetic tree exhibited a topology with high-resolution values at the clades. *Caroxylon* was initially considered a section of the genus *Salsola* [19,68,69], but later it was recognized as a separate genus [9,70]. The monophyly of *Caroxylon* reported in previous studies [6,20] remained consistent with the findings of this study. The assessment of the ML phylogenetic tree suggests that *C. passerinum* (syn. *C. gemmascens*) is the oldest species in the five analyzed taxa. In the four *Caraxylon* species reported in this study, *C. laricinum*, *C. dzhungaricum*, and *C. orientale* have formed a distinct subclade with a high bootstrap value (Figure 6). The fourth species, *C. nitrarium*, seems to have evolved from a common ancestor in the earlier stage of speciation, which is well agreed with the results obtained based on using universal DNA barcodes [6,9].

The robust support values obtained for the phylogenetic relationships inferred from plastid genome data closely mirrored those derived from nuclear gene data, suggesting the reliable resolution of phylogenetic relationships within this genus by plastid genome data. However, further plastid genome data are needed to comprehensively assess the phylogenetic relationships within the *Caroxylon* clade. This study represents the first attempt to evaluate phylogenetic relationships using genomic data in this genus and may serve as a valuable resource for future phylogenetic studies of the genus.

## 4. Materials and Methods

### 4.1. Plant Materials and DNA Extraction

Plant leaves of four *Caoxylon* species were collected from Kyzylorda (*C. orientale* and *C. nitrarium*), Almaty (*C. dzhungaricum*), and West Kazakhstan (*C. laricinum*) regions of Kazakhstan (Table 5). The voucher herbarium specimens of *C. orientale*, *C. nitrarium*, *C. dzhungaricum*, and *C. laricinum* were deposited in the Herbarium (AA) of the Institute of Botany and Phytointroduction under accession numbers AA0003263, AA0003264, AA0003265, and AA0003266, respectively. The collected fresh leaves were preserved in silica gel and used for DNA extraction. Total genomic DNA was extracted using the cetyltrimethylammonium bromide (CTAB) protocol [71], and DNA samples were stored at −80 °C until sequencing.

### 4.2. Genome Sequencing, Assembly, and Annotation

The total genomic DNA that passed quality control analysis was used for library preparation using the TruSeq Nano DNA Kit manufactured by Illumina Inc. (San Diego, CA, USA). The plastomes of four *Caroxylon* species were sequenced using an Illumina NovaSeq 6000 platform (Illumina Inc., USA), which was conducted at Macrogen Inc. (Seoul, Republic of Korea). FastQC (http://www.bioinformatics.babraham.ac.uk/projects/fastqc, accessed on 26 January 2024) was used to conduct initial quality control checks on raw sequence data coming from high throughput sequencing pipelines. The Trimmomatic 0.36 software [72] removed adapter sequences from the raw reads. The reads with a quality score over 20 were accepted as good-quality reads. Subsequently, the clean reads were assembled using the NOVOPlasty 4.3.3 program [73]. Further annotation of the assembled sequences was conducted using the published plastome of *Caroxylon passerinum* (MW192441) as a reference. The annotation of protein-coding, rRNA, and tRNA genes was performed using GeSeq [74] with manual corrections. The OrganellarGenomeDRAW 1.3.1 tool (OGDRAW) [75] generated a circular gene map of the four *Caroxylon* species. Finally, the annotated plastome sequences of four *Caroxylon* species were deposited into the GenBank.

### 4.3. Plastome Analysis by Sliding Window, Ka/Ks Calculation, IR Regions Contraction, and Expansion

To evaluate the nucleotide diversity (Pi) of the plastome sequences, we conducted sliding window analysis using DnaSP 6 software [76]. The window length was configured to 600 bp, with a step size of 200 bp. The synonymous (Ka), nonsynonymous (Ks), and Ka/Ks values of protein-coding genes in studied species were analyzed using DnaSP 6 software [76]. The contraction and expansion of the inverted repeat (IR) boundaries in *Caroxylon* species were visualized using IRscope software [77], with *C. passerinum* (MW192441) as the reference.

### 4.4. Repeat Sequence Analysis

Simple sequence repeats (SSR) were identified in four studied plastome sequences of *Caroxylon* species using the web-based simple sequence repeats finder MISA (https://webblast.ipk-gatersleben.de/misa/, accessed on 9 March 2024) tool [78]. The thresholds used were 8 repeat units for mononucleotide, 4 for dinucleotide and trinucleotide, and 3 for tetranucleotide, pentanucleotide, and hexanucleotide SSRs. Analysis of long repeats, including forward (F), reverse (R), and palindromic (P) repeats, was conducted using the REPuter program [79], accessible at https://bibiserv.cebitec.uni-bielefeld.de/reputer, accessed on 9 March 2024. This analysis utilized the following parameters: Hamming distance = 3 and a minimum repeat size of 30 base pairs. The Tandem Repeats Finder 4.09 (https://tandem.bu.edu/trf/trf.html, accessed on 9 March 2024) tool [80] was utilized to identify tandem repeats (T) with default settings.

### 4.5. Phylogenetic Analysis

Phylogenetic analysis was conducted using the newly sequenced four *Caroxylon* species, along with six related species and two outgroup species (*Suaeda glauca*, MK867773, and *Atriplex prostrata*, OR374024) obtained from GenBank. Phylogenetic trees were reconstructed using two sets of data: (1) nucleotide sequences from protein-coding genes and (2) nucleotide sequences of the entire plastid genome. Nucleotide sequences from 80 protein-coding genes were utilized, along with outgroups, to construct the phylogenetic tree using the Maximum Likelihood (ML) and Bayesian Inference (BI) methods. The ML phylogenetic tree was constructed using IQ-TREE 2.2.2.6 software [81], employing the best-fit model TVM + F + I + R3, selected based on the Bayesian Information Criterion (BIC). BI analysis was performed using MrBayes [82]. The generated trees were displayed using FigTree 1.4.4 (http://tree.bio.ed.ac.uk/software/figtree/, accessed on 10 March 2024).

## 5. Conclusions

The complete plastid genomes of four *Caroxylon* species were sequenced and annotated. The size of the plastid genomes varied from 150,777 in *C. nitrarium* to 151,307 in *C. orientale*. The comparative evaluation of the four plastid genomes indicated that they consisted of 133 genes in each species, including 80 protein-coding genes, 30 tRNA genes, and 4 rRNA genes. The regions *accD*, *atpF*, *matK*, *ndhF-ndhG*, *petB*, *rpl20-rpl22*, *rpoC2*, and *ycf3* were identified as the most divergent regions, with six out of ten genes in those regions being under positive selection. The analysis of four plastid genomes predicted the availability of 814 SSRs, with counts ranging from 198 in *C. nitrarium* to 208 in *C. dzhungaricum*. The ML phylogenetic tree confirmed the monophyletic origin of *Caroxylon*. The assessment of the dendrogram suggested that three *Caroxylon* species (*C. laricinum*, *C. dzhungaricum*, and *C. orientale*) have formed a distinct subclade with a robust genetic relationship. Thus, assessing the complete sequences of four plastid genomes in the genus provided highly informative data for future *Caroxylon* genetic studies.

## Figures and Tables

**Table 1:** Summary of plastid genome characteristics of *C. orientale*, *C. nitrarium*, *C. dzhungaricum*, and *C. laricinum*.

	*C. orientale*	*C. nitrarium*	*C. dzhungaricum*	*C. laricinum*
GenBank numbers	OR551471	OR552116	PP503423	PP503424
Genome size (bp)	151,307	150,777	151,148	151,115
LSC (bp)	83,693	83,329	83,706	83,678
SSC (bp)	18,266	18,986	18,996	18,999
IR (bp)	51,348	48,462	48,446	48,438
Number of total genes	133	133	133	133
Protein-coding genes	80	80	80	80
tRNAs	30	30	30	30
rRNAs	4	4	4	4
Total GC content (%)	36.69	36.84	36.68	36.71
LSC GC content (%)	34.69	34.86	34.67	34.69
SSC GC content (%)	29.68	30.33	28.80	29.82
IR GC content (%)	42.57	42.60	42.70	42.72

**Table 2:** Genes identified in plastomes of four *Caroxylon* species.

Category	Group of Genes	Name of Genes
Self-replication	Ribosomal RNA	*rrn4.5* (x2), *rrn5* (x2), *rrn16* (x2), *rrn23* (x2)
	Transfer RNA	*trnA-UGC* * (x2), *trnC-GCA*, *trnD-GUC*, *trnE-UUC*, *trnF-GAA*, *trnfM-CAU*, *trnG-GCC* *, *trnG-UCC*, *trnH-GUG*, *trnI-CAU* (x2), *trnI-GAU* * (x2), *trnK-UUU* *, *trnL-CAA* (x2), *trnL-UAA* *, *trnL-UAG*, *trnM-CAU*, *trnN-GUU* (x2), *trnP-UGG*, *trnQ-UUG*, *trnR-ACG* (x2), *trnR-UCU*, *trnS-GCU*, *trnS-GGA*, *trnS-UGA*, *trnT-GGU*, *trnT-UGU*, *trnV-GAC* (x2), *trnV-UAC* *, *trnW-CCA*, *trnY-GUA*
	Small subunit of ribosome	*rps2*, *rps3*, *rps4*, *rps7* (x2), *rps8*, *rps11*, *rps12* * (x2), *rps14*, *rps15*, *rps16* *, *rps18*, *rps19*
	Large subunit of ribosome	*rpl2* (x2), *rpl14*, *rpl16* *, *rpl20*, *rpl22*, *rpl23* (x2), *rpl32*, *rpl33*, *rpl36*
	RNA polymerase	*rpoA*, *rpoB*, *rpoC1* *, *rpoC2*
	Translation initiation factor	*infA*
Photosynthesis	ATP synthase	*atpA*, *atpB*, *atpE*, *atpF* *, *atpH*, *atpI*
	NADH dehydrogenase	*ndhA* *, *ndhB* * (x2), *ndhC*, *ndhD*, *ndhE*, *ndhF*, *ndhG*, *ndhH*, *ndhI*, *ndhJ*, *ndhK*
	Subunits of cytochrome	*petA*, *petB* *, *petD* *, *petG*, *petL*, *petN*
	Photosystem I	*psaA*, *psaB*, *psaC*, *psaI*, *psaJ*
	Photosystem II	*psbA*, *psbB*, *psbC*, *psbD*, *psbE*, *psbF*, *psbH*, *psbI*, *psbJ*, *psbK*, *psbL*, *psbM*, *psbN*, *psbT*, *psbZ*
	Rubisco	*rbcL*
Other genes	Maturase	*matK*
	Protease	*clpP* **
	Envelope membrane protein	*cemA*
	Subunit of acetyl-CoA-carboxylase	*accD*
	C-type cytochrome synthesis gene	*ccsA*
Genes of unknown function	Hypothetical chloroplast reading frames	*ycf1* (x2), *ycf2* (x2), *ycf3* **, *ycf4*, *ycf15* (x2)

* One-intron-containing genes; ** two-intron-containing genes; (x2) duplicated genes.

**Table 3:** Regions with highly variable sequences and Ka/Ks ratios of genes in plastid genomes of *Caroxylon* species.

Variable Region	Length	Variable Sites	Parsimony Informative Sites	Nucleotide Diversity	Analysed Genes	Ka/Ks Ratio
*accD*	723	103	36	0.05063	*accD*	0.900
*atpF*	771	75	35	0.04170	*atpF*	1.308
*matK*	606	96	37	0.05111	*matK*	1.813
*ndhF-ndhG*	606	101	44	0.05459	*ndhF* *ndhG*	0.600 0.476
*petB*	721	84	36	0.04252	*petB*	4.139
*rpl20-rpl22*	606	114	43	0.05711	*rpl20* *rpl22*	1.099 2.425
*rpoC2*	606	78	32	0.04141	*rpoC2*	0.476
*ycf3*	672	77	34	0.04185	*ycf3*	1.104

**Table 4:** Types and numbers of simple sequence repeats in the plastomes of four *Caroxylon* species.

Type	Repeat Unit	*C. orientale*	*C. nitrarium*	*C.dzhungaricum*	*C. laricinum*	Total (%)	%
Mono-	A/T	148	137	149	147	581 (97.81)	72.97
	C/G	3	4	3	3	13 (2.19)	
Di-	AC/GT	2	4	2	2	10 (6.45)	19.04
	AG/CT	15	15	15	15	60 (38.71)	
	AT/AT	20	25	21	19	85 (54.84)	
Tri-	AAG/CTT	-	2	2	2	6 (24)	3.07
	AAT/ATT	5	4	5	5	19 (76.00)	
Tetra-	AAAC/GTTT	1	1	1	1	4 (11.76)	4.18
	AAAG/CTTT	3	-	3	2	8 (23.53)	
	AAAT/ATTT	1	1	1	1	4 (11.76)	
	AAGG/CCTT	-	1	-	-	1 (2.94)	
	AATC/ATTG	-	-	1	-	1 (2.94)	
	AATT/AATT	2	2	2	2	8 (23.53)	
	ACCT/AGGT	2	2	2	2	8 (23.53)	
Penta-	AAAAG/CTTTT	1	-	1	1	3 (75.00)	0.49
	AAAGG/CCTTT	1	-	-	-	1 (25.00)	
Hexa-	AGCTCC/AGCTGG	-	-	-	2	2 (100.00)	0.25
Total		204	198	208	204	814 (100)	100

**Table 5:** Information on collected places of four *Caroxylon* species.

Species	Collected Place	GPS Coordinates
*C. orientale*	Kyzylorda region, Zhanakorgan district.	44.106111, 67.062778 150 m above sea level (m a. s. l.)
*C. nitrarium*	Kyzylorda region, Zhanakorgan district.	44.110833, 67.061111 150 m a. s. l.
*C. dzhungaricum*	Almaty region, Trans-Ili Alatau, left bank of the Charyn river.	43.249722, 78.898611 1210 m a. s. l.
*C. laricinum*	West Kazakhstan region, Burlinsky district, Bestau village.	51.254444, 53.093611 120 m a. s. l.

## Data Availability

Data are contained within the article.

## Supplementary Materials

The following supporting information can be downloaded at: https://www.mdpi.com/article/10.3390/plants13101332/s1, Supplementary File S1: SSRs list of *Caroxylon* species. Supplementary File S2: Phylogenetic tree based on complete plastid genome data.
