# Supplementary figures and images for: Characterization of the Plastid Genomes of Four Caroxylon Thunb. Species from Kazakhstan
Source: Plants (Basel). 2024 May 12;13(10):1332. doi: 10.3390/plants13101332 (PMC11124919; doi:10.3390/plants13101332)

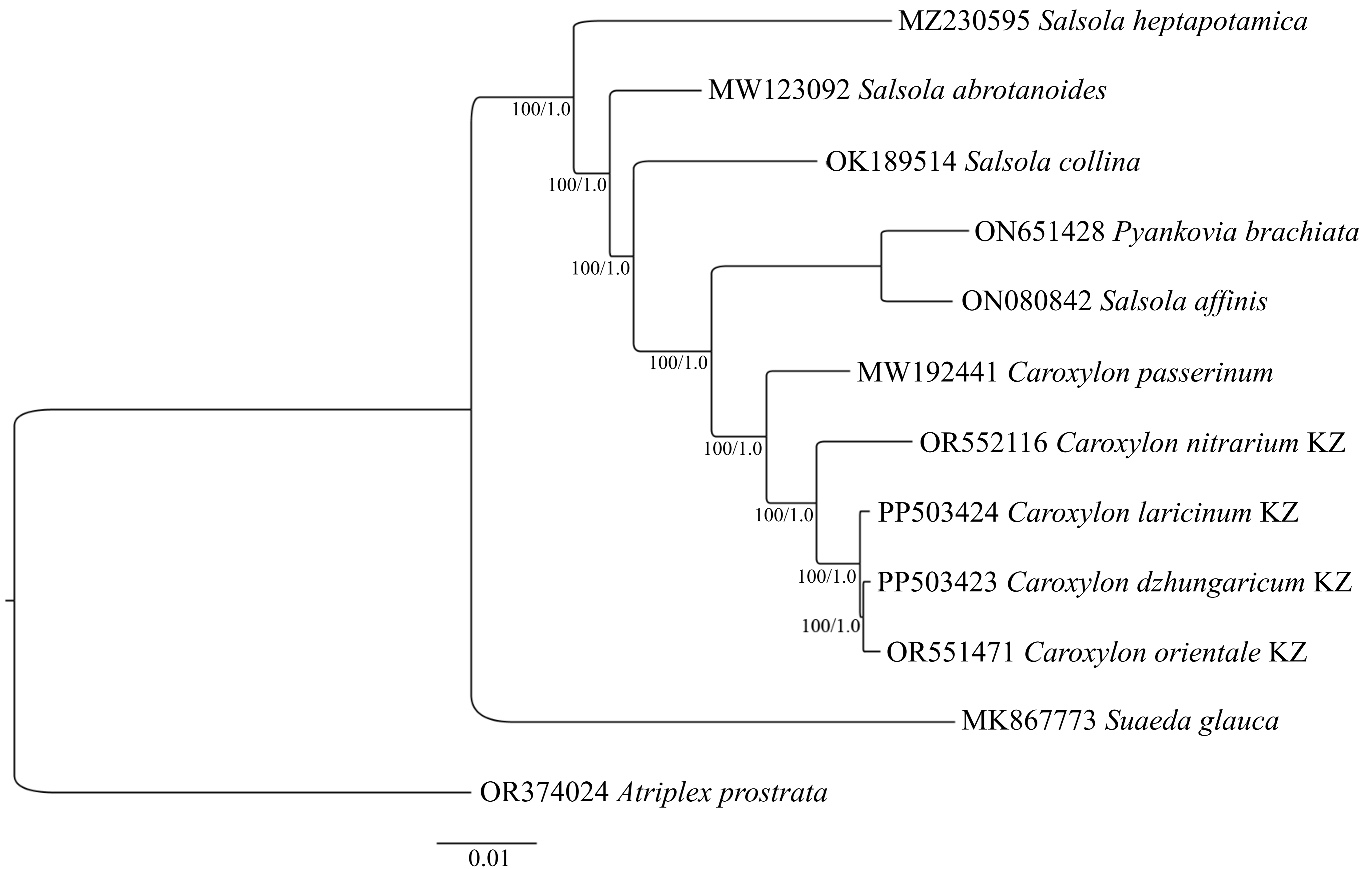

Supplement: Supplementary file 1 [file plants-13-01332-s001.zip › Supplementary File S2. Phylogenetic tree based on complete plastid genome data.pdf]
